# Systems-level analyses and clinical validation highlight CD53 as a diagnostic and prognostic marker in lung adenocarcinoma

**DOI:** 10.3389/fcell.2026.1806566

**Published:** 2026-06-11

**Authors:** Jun Chen, Weiming Tan, Yichen Wei, Zexian Han, Jichong Zhu, Kanglai Wei

**Affiliations:** 1 Department of Pathology, The Second Affiliated Hospital of Guangxi Medical University, Nanning, China; 2 The First Affiliated Hospital of Guangxi Medical University, Nanning, China

**Keywords:** biomarker, CD53, immune infiltration, lung adenocarcinoma, machine learning

## Abstract

**Background:**

Lung adenocarcinoma (LUAD) is the most common subtype of lung cancer, often diagnosed at advanced stages with poor prognosis. CD53, a tetraspanin involved in immune regulation, has an unclear role in LUAD.

**Methods:**

Five GEO LUAD transcriptomic datasets were integrated and batch-corrected. Differential expression and weighted gene co-expression network analyses identified LUAD-associated genes. Machine learning (Elastic Net) selected core predictive genes. Immune infiltration analysis, single-cell RNA-seq analysis, regulatory and drug–gene network construction, summary-data-based Mendelian randomization (SMR), and colocalization analyses were performed. Public TCGA-LUAD data were further used to evaluate the association between CD53 expression and clinicopathological characteristics. CD53 expression was clinically validated by immunohistochemistry (IHC) and PCR in 30 paired LUAD and adjacent normal tissue samples.

**Results:**

Six genes (BTK, CD163, CD53, F13A1, HCLS1, MS4A6A) were consistently downregulated in LUAD, with CD53 as a central immune-related candidate. CD53 expression positively correlated with monocytes and neutrophils, and negatively with naïve B cells, T follicular helper cells, plasma cells, and M0 macrophages. Single-cell RNA-seq analysis showed that CD53 was mainly localized to immune cell populations, particularly T cells, macrophages, and monocytes. SMR prioritized CD53 as a genetically associated candidate gene, whereas colocalization analysis did not support a shared causal variant between CD53 and LUAD. IHC and PCR both confirmed significantly lower CD53 expression in tumor tissues versus adjacent normal tissues, with low expression associated with poor prognosis. Public TCGA-LUAD analysis further showed that the CD53-low group had a higher proportion of deceased patients, and deceased patients exhibited lower CD53 expression than alive patients. Drug–gene network analysis predicted several candidate compounds potentially associated with CD53 and other model genes, providing hypothesis-generating clues for future experimental studies.

**Conclusion:**

CD53 is downregulated in bulk LUAD tissues, correlates with immune cell infiltration, and may serve as a candidate immune-microenvironment-associated diagnostic and prognostic biomarker. Its direct tumor-suppressive function remains to be determined in future mechanistic studies. These findings provide a basis for further mechanistic studies and future validation of CD53-related diagnostic, prognostic, and therapeutic hypotheses in LUAD.

## 1 Introduction

Lung adenocarcinoma (LUAD) is the most common histological subtype of lung cancer, accounting for approximately 40% of all cases (Zhang et al., 2023). Lung cancer remains the leading cause of cancer-related mortality worldwide, representing about 24% of cancer deaths. Due to the absence of typical symptoms in the early stages, the 5-year survival rate of patients diagnosed at advanced stages is less than 15%, highlighting the critical importance of early detection (Bray et al., 2024). Low-dose computed tomography (LDCT) has become the preferred screening modality for high-risk populations (Jonas et al., 2021). In recent years, genomic studies have revealed that driver gene mutations, including EGFR, ALK, and KRAS, play key roles in the initiation, progression, and prognosis of LUAD. These findings have provided essential insights for early diagnosis, molecular subtyping, and individualized targeted therapies, while also promoting the development of novel detection technologies such as liquid biopsy, enabling dynamic monitoring and precision intervention (Paliogiannis et al., 2022).

Bioinformatics offers multiple advantages in cancer research. The application of high-throughput sequencing and multi-omics technologies in oncology has generated massive amounts of data (Vitorino, 2024). Public databases such as TCGA and GEO enable the efficient integration of multi-omics datasets, facilitating the identification of genes closely associated with tumor development and providing valuable insights for precision oncology (Bi et al., 2021). In modern research, efficient data processing approaches have become increasingly essential, among which machine learning plays a pivotal role. Machine learning allows rapid analysis of large-scale datasets, particularly in medical applications, enabling the extraction of meaningful patterns from complex data (Alhumaidi et al., 2025). The strategic application of machine learning thus enhances the ability to process clinical and molecular data effectively, promoting the implementation of precision medicine (Kline et al., 2022).

CD53, a member of the tetraspanin family, is primarily involved in signal transduction and the formation of molecular complexes on the surface of immune cells (Dunlock et al., 2022). It plays a crucial role in immune cell activation and antigen presentation, and its low expression may impair antitumor immune responses (Dunlock, 2020). Previous research using single differential expression analyses has indicated that CD53 is downregulated in LUAD tissues (Jiang et al., 2022). However, no comprehensive studies have systematically investigated CD53 expression in LUAD across multiple datasets or validated its clinical relevance in patient samples.

The novelty of this study is reflected in several aspects. First, multiple datasets were integrated to minimize the bias inherent in single-cohort analyses. Second, a multi-dimensional approach was employed, including core gene screening in LUAD, machine learning, immune infiltration analysis, targeted drug prediction, and SMR analysis. Finally, clinical validation was performed using immunohistochemistry and PCR in 30 paired LUAD and adjacent normal tissue samples, thereby enhancing the translational relevance of the findings.

## 2 Materials and methods

### 2.1 Data collection and preprocessing

Five LUAD datasets with matched adjacent or normal lung tissues were downloaded from the GEO database, including GSE32863, GSE63459, GSE75037, GSE116959, and GSE118370. Data annotation and batch effect correction were performed using Perl, followed by sample labeling of LUAD and control groups in R. The GSE32863 and GSE63459 datasets were utilized for core gene screening, while the GSE75037, GSE116959, and GSE118370 datasets were employed for multi-dimensional analyses, including machine learning-based model construction, immune infiltration assessment, targeted drug prediction, and SMR analysis.

### 2.2 Identification of core genes

After batch effect correction, differential expression analysis was performed, and genes with adjusted P-value <0.05 were selected for further analysis. For WGCNA, genes with low expression variability were filtered out, and only genes with a standard deviation greater than 0.5 were retained. Sample clustering was performed to detect potential outliers, and samples were filtered using a static cut height of 20,000. The soft-thresholding power was selected using the pickSoftThreshold function in the WGCNA package across candidate powers from 1 to 20. The scale-free topology fit index threshold was set at 0.8, and the selected soft-thresholding power was β = 6. An adjacency matrix was then constructed and transformed into a topological overlap matrix. Gene modules were identified using the dynamic tree cut algorithm with deepSplit = 2, pamRespectsDendro = FALSE, and a minimum module size of 60. Modules with highly similar eigengenes were merged using a module eigengene dissimilarity threshold of 0.25. Module–trait correlations were calculated between module eigengenes and LUAD/control status, and the LUAD-associated module was selected for downstream analysis.

The intersection of differentially expressed genes and LUAD-associated co-expression module genes was subjected to GO and KEGG enrichment analyses. Protein–protein interaction networks were constructed using the STRING database, and core genes within the networks were identified using Cytoscape and its plug-ins.

### 2.3 Machine learning modeling

In the GSE75037, GSE116959, and GSE118370 datasets, the training cohorts were used to construct predictive models employing various machine learning algorithms, including Stepglm, glmBoost, GBM, LDA, and Enet, either individually or in combination. Leveraging the strengths of different algorithms, the optimal model was selected based on the AUC values obtained in both the training and validation cohorts.

The diagnostic performance of the optimal model was further evaluated using multiple metrics, including AUC, accuracy, sensitivity, specificity, positive predictive value, negative predictive value, F1 score, and Brier score (Supplementary Table S1). LUAD samples were defined as the positive class, and control samples were defined as the negative class. Predicted probabilities were converted into binary classifications using a threshold of 0.5. Calibration performance was assessed using calibration curves based on predicted probabilities and observed LUAD proportions.

### 2.4 Post-model analyses of selected genes

Following the construction of the optimal diagnostic model, downstream analyses were performed to further explore the biological and clinical relevance of the selected genes. Differential expression and immune infiltration analyses were conducted to evaluate associations between model genes and immune cell populations. Regulatory networks, including transcription factor–gene and drug–gene interaction networks, were constructed to investigate potential regulatory mechanisms and therapeutic targets. Additionally, summary-data-based Mendelian randomization (SMR) was performed by integrating GWAS and eQTL data, and Manhattan plots were generated to identify significant associations. Finally, Kaplan–Meier survival analysis was conducted using the Kaplan–Meier Plotter database to assess the prognostic significance of individual model genes.

### 2.5 Single-cell RNA-seq analysis

To further investigate tumor heterogeneity and the cell-type-specific distribution of CD53 in the LUAD microenvironment, we performed single-cell RNA-seq analysis using the public LUAD single-cell RNA-seq dataset GSE149655, which included two normal lung tissue samples and two LUAD tissue samples. After quality control, high-quality cells were retained for downstream analysis. Single-cell data processing was performed using the Seurat package in R. Cells with poor quality were filtered according to the number of detected genes, total transcript counts, and mitochondrial gene percentage. After normalization and identification of highly variable genes, principal component analysis was performed. The first 20 principal components were selected for downstream clustering based on JackStraw analysis. Cells were clustered and visualized using t-distributed stochastic neighbor embedding (t-SNE). Major cell types were annotated according to canonical marker genes and known cell-type signatures. CD53 expression was then visualized across annotated cell populations and between normal and tumor samples using t-SNE feature plots, violin plots, and dot plots.

### 2.6 Clinical validation

Paraffin-embedded tissue specimens from 30 LUAD patients and their matched adjacent normal tissues were collected and sectioned. Immunohistochemistry (IHC) staining was performed using a recombinant CD53 monoclonal antibody (Wuhan Sanying Company, Cat. No. 84837-1-RR) following the manufacturer’s instructions. Stained slides were carefully examined under an inverted microscope, and representative images were captured using appropriate imaging equipment. All IHC images were quantitatively analyzed using ImageJ software to assess CD53-positive expression. CD53 expression was quantified as the percentage of CD53-positive cells among the total counted cells. Statistical comparisons of CD53-positive expression between paired LUAD tissues and matched adjacent normal tissues were conducted using paired t-tests or Wilcoxon signed-rank tests, as appropriate.

Additionally, PCR analysis was performed in 30 paired LUAD and adjacent normal tissue samples to measure the mRNA expression levels of CD53, providing complementary validation to the IHC findings. Total RNA was extracted from tumor and adjacent normal tissues, followed by reverse transcription into cDNA. Quantitative PCR was performed to assess CD53 mRNA expression, with GAPDH used as the internal reference. Relative expression levels were calculated using the 2^-ΔΔCt method. Statistical comparisons between paired tumor and adjacent normal tissues were performed using paired t-tests or Wilcoxon signed-rank tests, as appropriate.

### 2.7 Public database-based clinicopathological analysis

Because complete clinicopathological information was not available for our in-house validation cohort, we further evaluated the clinicopathological relevance of CD53 using the TCGA-LUAD cohort. CD53 mRNA expression data and corresponding clinicopathological information were obtained from public TCGA-LUAD resources. Primary LUAD tumor samples were retained for analysis. Patients were divided into CD53-low and CD53-high groups according to the median CD53 mRNA expression level in tumor tissues. Associations between CD53 expression groups and available clinicopathological parameters, including age, sex, pathological stage, T stage, N stage, M stage, and vital status, were analyzed using the chi-square test or Fisher’s exact test, as appropriate. Differences in continuous CD53 expression between clinical subgroups were evaluated using the Mann–Whitney U test. A two-sided P value <0.05 was considered statistically significant.

## 3 Results

### 3.1 Overview of the study workflow and batch-corrected GEO datasets

**FIGURE 1:**
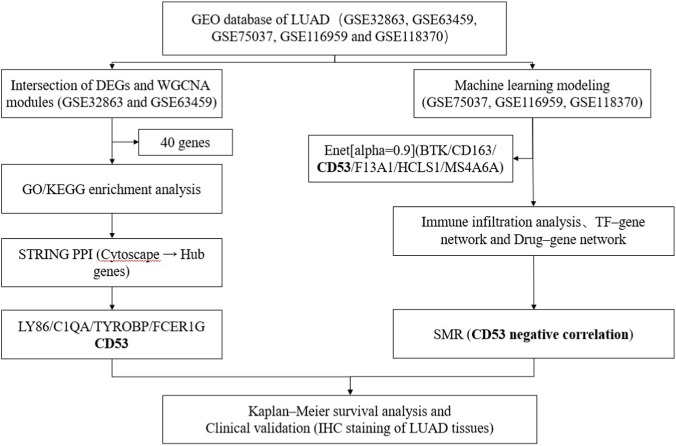
Study design and analytical workflow of multi-cohort LUAD transcriptomic integration, machine learning, immune infiltration, and clinical validation.

**FIGURE 2:**
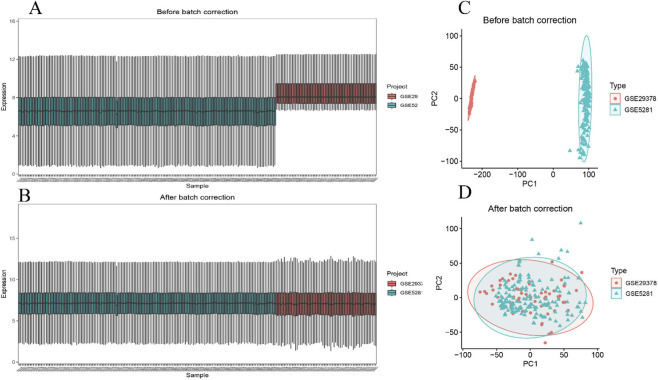
Boxplots and principal component analysis (PCA) demonstrating batch effect correction and comparability across merged GEO LUAD datasets. **(A)** Boxplot before batch correction. **(B)** Boxplot after batch correction. **(C)** PCA plot before batch correction. **(D)** PCA plot after batch correction.

The overall study design and analytical workflow are summarized in Figure 1. Boxplots and principal component analysis (PCA) of the merged datasets are presented in Figure 2. These analyses demonstrate that batch effects among the datasets were effectively corrected, ensuring comparability across samples.

### 3.2 Identification of LUAD-associated DEGs and WGCNA modules

**FIGURE 3:**
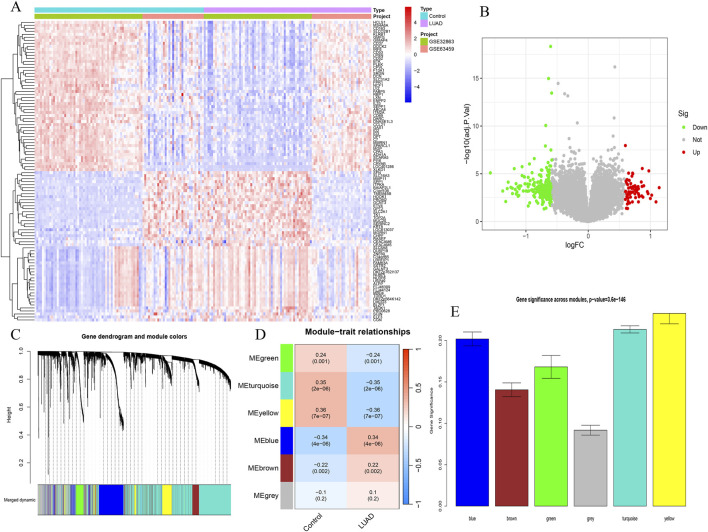
Identification of differentially expressed genes and WGCNA modules associated with LUAD. **(A)** Heatmap of the top 50 differentially expressed genes between LUAD and control samples in GSE32863 and GSE63459. **(B)** Volcano plot showing all differentially expressed genes. Red and blue dots indicate significantly upregulated and downregulated genes, respectively, according to the predefined adjusted P-value threshold. **(C)** Sample clustering and WGCNA analysis used to identify co-expression modules. **(D)** Module–trait relationship heatmap showing correlations between WGCNA modules and LUAD/control status. The color scale represents the strength and direction of the correlation, and the values in each cell indicate the correlation coefficient and corresponding P-value. **(E)** Gene significance across WGCNA modules. The yellow module showed the highest gene significance and was selected as the LUAD-associated module for subsequent intersection and downstream analyses.

A total of 266 differentially expressed genes (DEGs) were identified. Figure 3A presents a heatmap of the top 50 DEGs in GSE32863 and GSE63459, illustrating their expression patterns in LUAD and control samples, with blue indicating low expression and red indicating high expression. Figure 3B shows the corresponding volcano plot of all DEGs. The WGCNA co-expression analysis is depicted in Figure 3C. The soft-thresholding power was selected based on the scale-free topology criterion, and module detection was performed using the dynamic tree cut algorithm with a minimum module size of 60 and a module merging threshold of 0.25. The module–trait relationship heatmap further showed that the yellow module had a strong association with LUAD status, as indicated by the module–trait correlation coefficients and corresponding P-values (Figure 3D). After gene clustering, LUAD-related genes were predominantly grouped in the yellow module, as shown in Figure 3E. Genes within this yellow module were subsequently extracted for further downstream analyses.

### 3.3 Functional enrichment and PPI network analysis identify immune-related hub genes

**FIGURE 4:**
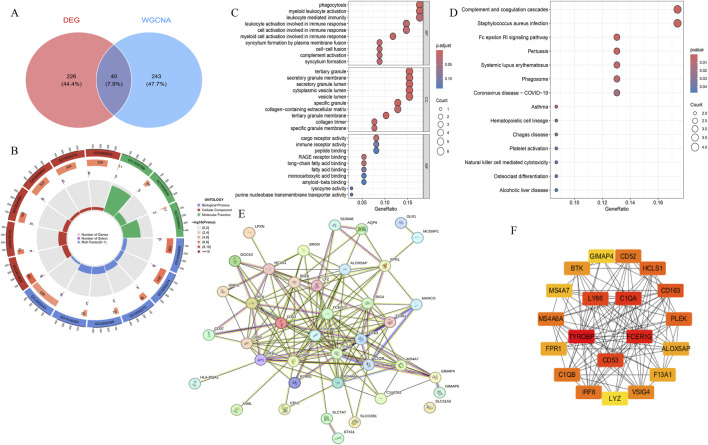
Functional annotation and PPI network of overlapping LUAD-associated genes: **(A)** Venn diagram of DEGs and WGCNA module genes. **(B)** Circular plot of GO term enrichment. **(C)** GO enrichment analysis. **(D)** KEGG pathway enrichment analysis. **(E)** Protein–protein interaction (PPI) network. **(F)** Hub gene identification within the PPI network, including CD53. The enrichment results suggest that the overlapping genes are mainly involved in innate immune activation, myeloid-cell function, phagocytosis, and complement-related inflammatory responses.

The intersection of DEGs in LUAD and disease-related genes identified by WGCNA yielded 40 overlapping genes (Figure 4A). In Figure 4B, the outermost circle represents GO term IDs, the second circle indicates the number of genes associated with each GO term, and the third circle shows the number of overlapping genes enriched in each GO term. Figure 4C presents the GO enrichment analysis of the overlapping genes, revealing that they were primarily involved in phagocytosis, myeloid leukocyte activation, tertiary granule, secretory granule membrane, and cargo receptor activity. Figure 4D shows KEGG enrichment analysis, indicating significant enrichment in complement and coagulation cascades and infection-related immune-response pathways.

These enrichment results indicate that the overlapping LUAD-associated genes were mainly involved in innate immune activation and myeloid-cell-related biological processes. In particular, enrichment of phagocytosis, myeloid leukocyte activation, secretory granule membrane, and tertiary granule-related terms suggests that the identified gene set may participate in macrophage/monocyte activation, neutrophil degranulation, antigen handling, and inflammatory remodeling within the LUAD microenvironment. KEGG enrichment in complement and coagulation cascades further suggests that these genes may be linked to inflammatory amplification, immune-cell recruitment, and tumor–stroma interactions. Therefore, the enrichment pattern supports the immune-related nature of the CD53-containing hub-gene module and provides a mechanistic rationale for subsequent immune infiltration and single-cell analyses.

Figure 4E illustrates the protein–protein interaction (PPI) network among the overlapping genes, with edge colors representing different types of evidence. Using Cytoscape plug-ins, hub genes within the network were identified (Figure 4F), which included LY86, C1QA, TYROBP, FCER1G, and CD53.

### 3.4 Machine learning identifies six downregulated diagnostic genes and prioritizes CD53

**FIGURE 5:**
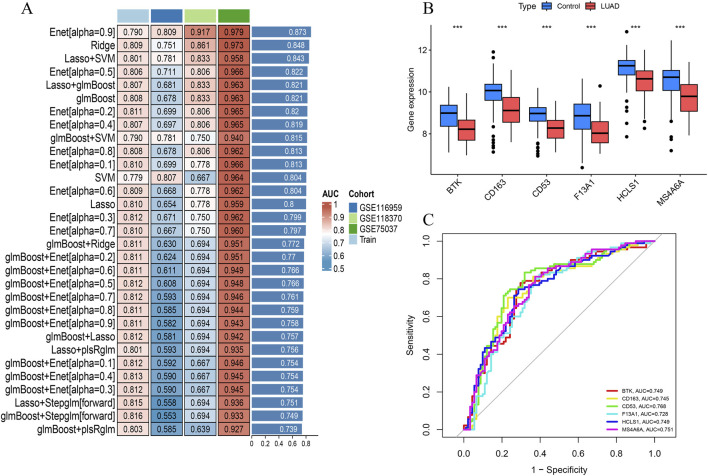
Machine learning-based identification of core LUAD genes: **(A)** Elastic Net model performance across training and validation datasets (AUC values). **(B)** Expression patterns of six model genes (BTK, CD163, CD53, F13A1, HCLS1, MS4A6A) in LUAD. **(C)** Diagnostic performance (AUC) of individual model genes.

To validate our gene expression findings, we used GSE75037, GSE116959, and GSE118370 as control datasets and applied multiple machine learning algorithms for feature selection. As shown in Figure 5A, the Elastic Net algorithm (α = 0.9) achieved the optimal performance, with an AUC of 0.790 in the training set and AUCs of 0.809, 0.917, and 0.979 in the three validation datasets, yielding an average AUC of 0.873. This approach identified six genes—BTK, CD163, CD53, F13A1, HCLS1, and MS4A6A—which were all downregulated in LUAD (Figure 5B). The diagnostic performance of each individual gene was further evaluated, with respective AUCs of 0.749, 0.745, 0.768, 0.728, 0.749, and 0.751 (Figure 5C). To further evaluate model performance beyond AUC, we calculated additional classification metrics using a probability threshold of 0.5. As shown in Supplementary Table S1, the Elastic Net model achieved an accuracy of 0.740, sensitivity of 0.689, and specificity of 0.791 in the training cohort. In the three external validation cohorts, the model showed accuracies of 0.618, 0.750, and 0.934; sensitivities of 0.544, 0.833, and 0.880; and specificities of 1.000, 0.667, and 0.988 in GSE116959, GSE118370, and GSE75037, respectively. In the pooled validation cohort, the model achieved an AUC of 0.901, accuracy of 0.837, sensitivity of 0.747, and specificity of 0.970. Calibration curve analysis further showed the agreement between predicted probabilities and observed LUAD proportions in the training and pooled validation cohorts (Supplementary Figure S1). Notably, CD53 was consistently identified as a core gene exhibiting abnormal expression in LUAD across the machine learning validation datasets, highlighting its potential central role in the disease.

Although all six model genes showed consistent downregulation in LUAD and contributed to the diagnostic model, CD53 was selected as the main focus of subsequent analyses based on several considerations. First, CD53 showed the highest single-gene diagnostic AUC among the six model genes, suggesting relatively favorable individual diagnostic performance. Second, CD53 was identified as one of the hub genes in the immune-related PPI network derived from the overlapping DEG–WGCNA gene set. Third, CD53 showed strong and biologically interpretable associations with immune-cell infiltration, especially monocytes and neutrophils, which was consistent with the innate immune and myeloid-cell-related enrichment results. Fourth, CD53 was further supported by SMR analysis, survival analysis, clinical validation, public TCGA-LUAD clinicopathological analysis, and single-cell localization analysis. Therefore, we prioritized CD53 for in-depth mechanistic interpretation, while the other five genes were retained in the model-level, immune infiltration, regulatory network, and drug–gene network analyses.

### 3.5 Model genes are associated with immune-cell infiltration, with CD53 showing prominent immune relevance in LUAD

**FIGURE 6:**
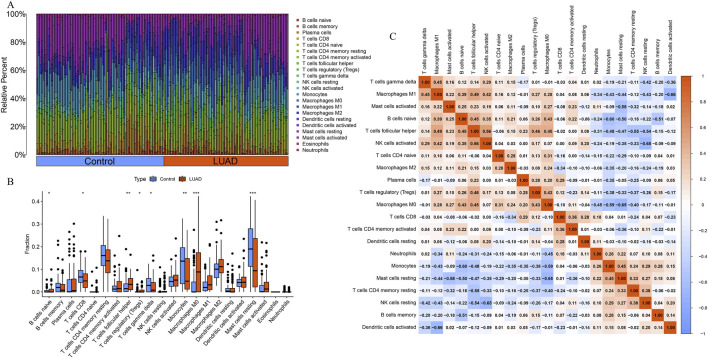
Immune infiltration analysis in LUAD using CIBERSORT: **(A,B)** Proportions of immune cell types in tumor versus normal tissues. **(C)** Correlation heatmap among immune cell populations.

**FIGURE 7:**
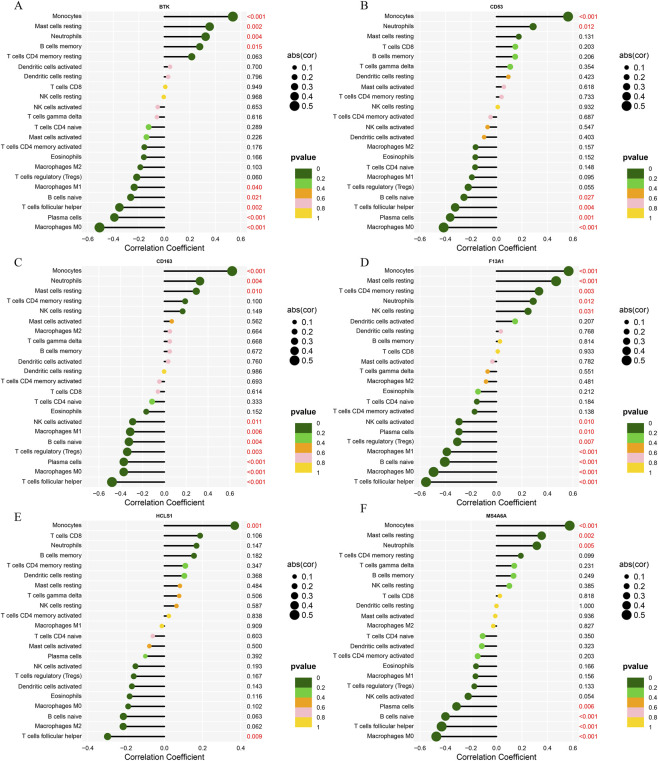
Correlation between model gene expression and immune cell infiltration: **(A–F)** Immune infiltration patterns for BTK, CD163, CD53, F13A1, HCLS1, and MS4A6A.

We next evaluated the relationship between model genes and immune-cell infiltration using the CIBERSORT algorithm. Immune infiltration was evaluated using the CIBERSORT algorithm (Figures 6A,B). The results revealed that naïve B cells, follicular helper T cells, regulatory T cells (Tregs), and M0 macrophages were significantly enriched in LUAD tissues. In contrast, CD8^+^ T cells, gamma delta T cells, monocytes, and resting mast cells were present at lower proportions, with statistically significant differences. Figure 6C illustrates the correlations among immune cell types, with red indicating positive correlations and blue indicating negative correlations. Figures 7A–F present the immune infiltration analysis of BTK, CD163, CD53, F13A1, HCLS1, and MS4A6A. Notably, CD53 expression was positively correlated with monocytes and neutrophils, with statistically significant differences. In contrast, CD53 expression was negatively correlated with naïve B cells, follicular helper T cells, plasma cells, and M0 macrophages, also showing statistically significant differences.

### 3.6 Regulatory and drug–gene network analyses generate candidate therapeutic hypotheses

**FIGURE 8:**
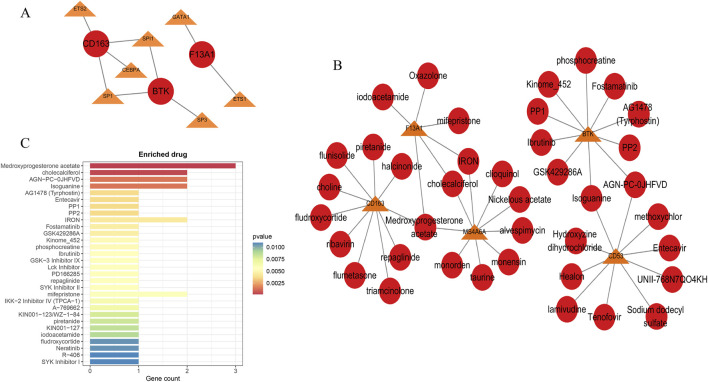
Regulatory and drug–gene network analysis. **(A)** Gene regulatory network of model genes highlighting central nodes. **(B)** Drug–gene interaction network showing predicted compound–gene associations. **(C)** Representative predicted compounds associated with multiple model genes. These compounds should be interpreted as computationally predicted candidates and require further experimental validation.

Based on the model genes identified by machine learning, we further constructed a gene regulatory network (Figure 8A). The results demonstrated that BTK, CD163, and F13A1 occupied central nodes within the network, suggesting that these genes may participate in LUAD-associated molecular regulation through potential gene–gene interactions. Subsequently, a drug–gene interaction network was constructed to explore possible compound–gene associations at the bioinformatics level (Figure 8B). The analysis showed that the model genes were linked to several predicted or database-derived compounds. For example, medroxyprogesterone acetate has been discussed in previous systematic evidence in relation to hormone-related cancer risk, suggesting that predicted compounds identified through drug–gene network analysis should be interpreted cautiously and require further validation before any clinical implication can be inferred (Zürcher et al., 2024, Figure 8C). Importantly, these compounds should be interpreted only as computationally predicted candidates rather than validated therapeutic agents for LUAD. Their biological effects, target specificity, and potential anti-tumor efficacy require further validation through *in vitro* and *in vivo* experiments.

### 3.7 SMR, colocalization, and survival analyses prioritize CD53 as a genetically associated and prognostic candidate without establishing causality

We first organized the exposure data, which were obtained from the GWAS database (https://gwas.mrcieu.ac.uk/) by retrieving the exposure profiles of the model genes based on their IDs. The outcome data for LUAD were downloaded from the FinnGen database (https://www.finngen.fi/en/access_results). Subsequently, colocalization analysis was performed to evaluate whether the model genes shared a common genetic basis with LUAD. As shown in Table 1, no gene exhibited a high posterior probability of colocalization (PP4 < 0.75), with the highest observed value being 0.2398 for CD53. These findings indicate that the current colocalization analysis does not provide strong evidence that the model genes influence LUAD development through shared causal genetic variants.

**TABLE 1:** Colocalization analysis of model genes and LUAD GWAS loci, showing posterior probabilities (PP4) for shared genetic variants.

Symbol	PP.H0	PP.H1	PP.H2	PP.H3	PP.H4
MS4A6A	0	0.976961	0	0.007606	0.015432
F13A1	0	0.934623	0	0.027726	0.037651
CD53	1.04E-104	0.703044	8.53E-106	0.057188	0.239768
CD163	2.85E-25	0.950869	4.58E-27	0.015248	0.033882
HCLS1	0	0.212733	0	0.642704	0.144563

**FIGURE 9:**
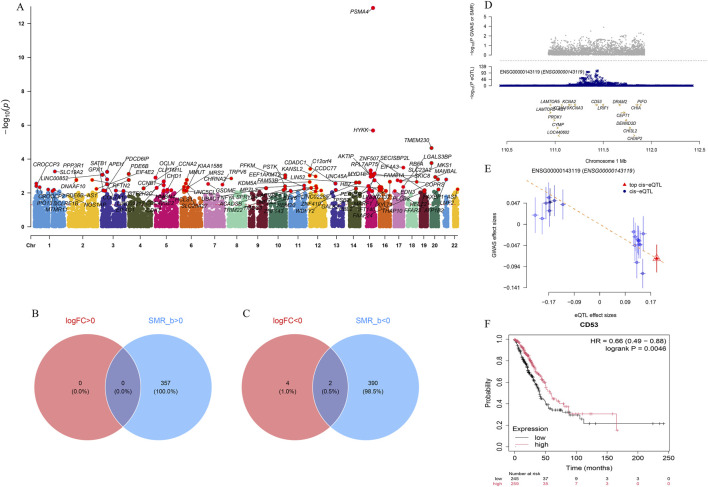
Genetic association, SMR, colocalization-related interpretation, and survival analysis of model genes in LUAD. **(A)** Manhattan plot showing disease-associated genes identified by SMR analysis based on chromosomal positions and association P-values. **(B)** Intersection analysis between upregulated model genes and SMR-significant genes. **(C)** Intersection analysis between downregulated model genes and SMR-significant genes, identifying CD53 and HCLS1 as candidate downregulated genes associated with LUAD. **(D)** Single-gene regional association plot showing the chromosomal position and association signals of CD53 at the gene-expression and disease-association levels. **(E)** SMR effect plot showing the association direction between genetically predicted CD53 expression and LUAD. **(F)** Kaplan–Meier survival curve comparing overall survival between patients with high and low CD53 expression. Patients were stratified according to the cutoff used in the Kaplan–Meier Plotter platform. Survival differences were evaluated using the log-rank test, and hazard ratio with 95% confidence interval and P-value are shown in the plot. Statistical significance was defined as P < 0.05.

After organizing the LUAD outcome data, we performed SMR analysis and identified a total of 749 disease-associated genes. The Manhattan plot based on chromosomal positions and P-values (Figure 9A) highlighted several genes significantly associated with LUAD. We then intersected the model genes with the SMR-significant genes. As shown in Figure 9B, no high-risk genes were identified among the upregulated genes, whereas two potential high-risk genes, CD53 and HCLS1, were detected among the downregulated genes (Figure 9C).

Subsequent single-gene analyses further supported these findings. In Figure 9D, the x-axis indicates the chromosomal position of each gene, while the y-axis represents the P-values for both gene expression and disease association. CD53 exhibited significant associations at both the gene-expression and disease-association levels. Moreover, Figure 9E suggested a negative association between genetically predicted CD53 expression and LUAD. However, given the lack of strong colocalization evidence, this result should be interpreted as a genetic association rather than proof of causality. To validate these findings, we analyzed CD53 using the Kaplan–Meier Plotter platform (https://kmplot.com/analysis/). Kaplan–Meier survival analysis further showed that patients with low CD53 expression had significantly poorer survival than those with high CD53 expression, based on the grouping cutoff implemented in the Kaplan–Meier Plotter platform and log-rank test comparison (Figure 9F).

### 3.8 Clinical validation confirms reduced CD53 expression in LUAD tissues

**FIGURE 10:**
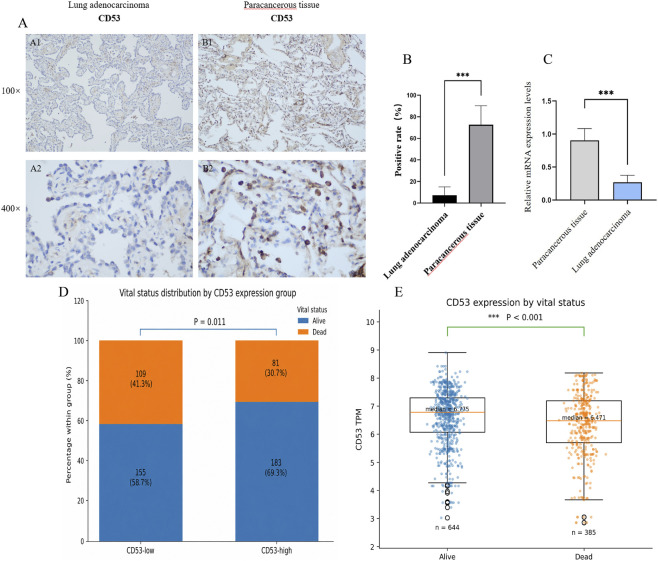
Clinical and public-cohort validation of CD53 expression and prognostic relevance in LUAD. **(A)** Representative immunohistochemical staining images showing stronger CD53 expression in adjacent normal tissues and reduced CD53 staining in LUAD tissues. **(B)** Quantitative analysis of CD53-positive cell percentage in 30 paired LUAD and adjacent normal tissue samples. **(C)** PCR analysis showing lower CD53 mRNA expression in 30 paired LUAD tissues than in adjacent normal tissues. **(D)** Distribution of vital status between the CD53-low and CD53-high groups in the TCGA-LUAD cohort. Patients were dichotomized according to the median CD53 mRNA expression level. The CD53-low group contained a higher proportion of deceased patients than the CD53-high group. **(E)** Comparison of CD53 mRNA expression between alive and deceased patients in the TCGA-LUAD cohort. CD53 expression was significantly lower in deceased patients than in alive patients.

To further validate the findings from bioinformatics and statistical analyses, we expanded the clinical validation cohort and performed IHC assays on tumor tissues and matched adjacent normal tissues obtained from 30 pathologically confirmed LUAD patients. The results demonstrated that CD53 exhibited stronger positive staining in adjacent normal tissues, whereas its staining intensity and positive-cell percentage were markedly reduced in LUAD tissues (Figures 10A,B). PCR analysis in 30 paired LUAD and adjacent normal tissue samples further confirmed that CD53 mRNA expression was significantly lower in LUAD tissues than in adjacent normal tissues (Figure 10C). These findings are consistent with our previous results derived from machine learning and multi-omics analyses, reinforcing the evidence that CD53 is downregulated in LUAD tissues and may serve as a candidate immune-associated biomarker rather than a validated tumor suppressor.

To further evaluate the clinicopathological relevance of CD53, we performed a supplementary analysis using the TCGA-LUAD cohort. Patients were divided into CD53-low and CD53-high groups according to the median CD53 mRNA expression level in primary LUAD tumor tissues. As shown in Table 2, CD53 expression was significantly associated with age and vital status. In particular, the CD53-low group contained a higher proportion of deceased patients than the CD53-high group (109/264 [41.3%] vs. 81/264 [30.7%], P = 0.011; Figure 10D). Consistently, continuous-expression analysis showed that CD53 expression was significantly lower in deceased patients than in alive patients (median: 6.389 vs. 6.705, P = 0.003; Figure 10E). In addition, lower CD53 expression tended to be associated with more advanced pathological stage and higher T stage, although these associations did not reach statistical significance. These findings provide additional public-cohort evidence supporting the clinicopathological and prognostic relevance of CD53 in LUAD.

**TABLE 2:** Association between CD53 expression and clinicopathological characteristics in the TCGA-LUAD cohort.

Variable	Category	Total	CD53-low	CD53-high	P Value
Age	<65	230	131	99	**0.005**
	≥65	279	124	155	​
Sex	Female	285	132	153	0.067
	Male	243	132	111	​
Pathologic stage	Stage I–II	410	196	214	0.078
	Stage III–IV	110	63	47	​
T stage	T1–T2	458	223	235	0.092
	T3–T4	67	40	27	​
N stage	N0	341	165	176	0.162
	N1–N3	173	95	78	​
M stage	M0	357	181	176	0.368
	M1	25	15	10	​
Vital status	Alive	338	155	183	**0.011**
	Dead	190	109	81	​

Bold values indicate statistical significance (P < 0.05).

### 3.9 Single-cell analysis revealed the immune-cell-specific distribution of CD53 in LUAD

**FIGURE 11:**
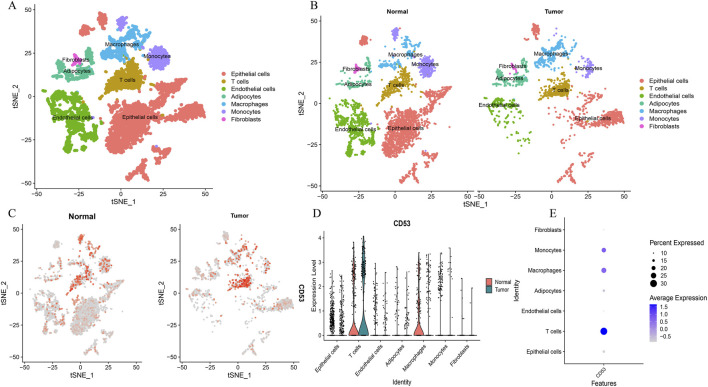
Single-cell RNA-seq analysis of CD53 distribution in the LUAD microenvironment. **(A)** t-SNE visualization of annotated cell populations in the integrated LUAD single-cell dataset, including epithelial cells, T cells, endothelial cells, adipocytes, macrophages, monocytes, and fibroblasts. **(B)** t-SNE visualization stratified by tissue type, showing the distribution of major cell populations in normal lung and LUAD samples. **(C)** t-SNE feature plots showing CD53 expression in normal and tumor samples. CD53 expression was mainly localized to immune-cell-enriched regions. **(D)** Violin plot showing CD53 expression across annotated cell types and tissue groups. CD53 was predominantly expressed in T cells, macrophages, and monocytes, with relatively low expression in epithelial and stromal cell populations. **(E)** Dot plot showing the average expression level and percentage of CD53-expressing cells across annotated cell types.

To address tumor heterogeneity and clarify the cellular origin of CD53 expression in LUAD, we further performed single-cell RNA-seq analysis using two normal lung samples and two LUAD samples. After quality control, dimensionality reduction, and clustering, the integrated single-cell atlas identified several major cell populations within the LUAD microenvironment, including epithelial cells, T cells, macrophages, monocytes, fibroblasts, endothelial cells, and adipocytes (Figure 11A). When cells were stratified by tissue type, normal and tumor samples showed different cellular distributions, suggesting substantial microenvironmental heterogeneity between normal lung and LUAD tissues (Figure 11B).

We next examined the cell-type-specific distribution of CD53. The t-SNE feature plots showed that CD53 expression was mainly concentrated in immune-cell-enriched regions rather than being broadly distributed across epithelial cell clusters (Figure 11C). Violin plot analysis further showed that CD53 was predominantly expressed in T cells, macrophages, and monocytes, whereas its expression was relatively low in epithelial cells, fibroblasts, endothelial cells, and adipocytes (Figure 11D). Consistently, dot plot analysis confirmed that T cells and myeloid-lineage cells exhibited the highest average expression and expression proportion of CD53 among the annotated cell populations (Figure 11E).

These single-cell findings indicate that CD53 is primarily an immune-cell-associated gene in the LUAD microenvironment. Therefore, the reduced CD53 expression observed in bulk transcriptomic, IHC, and PCR analyses may partly reflect alterations in immune-cell composition or immune microenvironmental remodeling in LUAD tissues, rather than tumor-cell-intrinsic downregulation alone.

## 4 Discussion

In this study, we integrated multi-cohort transcriptomic data, machine learning–based feature selection, immune infiltration profiling, regulatory and drug–gene network construction, and genetic epidemiology analyses to identify and validate LUAD-associated genes. Using elastic net modeling and cross-dataset validation, six robust genes (BTK, CD163, CD53, F13A1, HCLS1, and MS4A6A) were consistently downregulated, with CD53 emerging as a core immune-related candidate, validated experimentally via IHC and PCR in clinical samples.

The remaining five model genes also have biological plausibility in LUAD-related immune remodeling. BTK is a key kinase involved in B-cell receptor signaling and myeloid-cell activation, and may reflect immune signaling alterations within the tumor microenvironment. CD163 is a well-known marker of macrophage polarization and tumor-associated macrophages, supporting the myeloid immune signature observed in this study. F13A1 has been reported as a macrophage-associated coagulation and extracellular matrix-related gene, consistent with the enrichment of complement/coagulation and stromal-remodeling pathways. HCLS1 participates in hematopoietic cell signaling and immune-cell activation, whereas MS4A6A is closely related to myeloid-lineage immune regulation. Therefore, the six-gene model as a whole appears to represent an immune- and myeloid-enriched transcriptional signature in LUAD. Nevertheless, CD53 was selected for focused validation because it showed relatively favorable individual diagnostic performance, clear immune-cell associations, supportive SMR and survival results, and consistent clinical and single-cell evidence.

Although prior LUAD studies and public resources provide limited insights on CD53, our multi-omic approach highlights its role in tumor immunity (Hao et al., 2022). Human Protein Atlas data show CD53 expression is moderate in macrophages but low in alveolar epithelial cells, suggesting a basal immune function rather than direct epithelial involvement (https://www.proteinatlas.org/ENSG00000143119-CD53?utm_source=chatgpt.com). Previous immune prognostic signatures rarely included CD53, underscoring the novelty of our findings (Li et al., 2023a).

CD53, a tetraspanin broadly expressed on immune cells, modulates T cell activation, regulates neutrophil extracellular trap formation, and influences host defense against infections (Hsu et al., 2025). Its role in autoimmune, inflammatory, and infectious contexts highlights a broader immunoregulatory function, supporting the relevance of CD53 in shaping tumor-immune interactions in LUAD (Higgins et al., 2023).

Immune infiltration analysis revealed that CD53 expression positively correlates with monocytes and neutrophils, and negatively with naïve B cells, T follicular helper cells, plasma cells, and M0 macrophages, suggesting a role in shaping the tumor immune microenvironment and influencing antitumor immunity (Wang et al., 2022). The enrichment analysis further supports this immune-microenvironment-centered interpretation. The CD53-containing LUAD-associated gene set was enriched in phagocytosis, myeloid leukocyte activation, secretory granule membrane, tertiary granule-related processes, and complement/coagulation pathways. These biological processes are closely related to innate immune-cell activation, macrophage and monocyte function, neutrophil degranulation, inflammatory mediator release, and immune-cell recruitment. In the context of LUAD, such pathways may contribute to remodeling of the tumor immune microenvironment rather than reflecting tumor epithelial programs alone. Therefore, the enrichment results suggest that CD53 may be embedded in an immune-regulatory module involving myeloid activation, phagocytic activity, and complement-associated inflammatory signaling. This interpretation is consistent with our CIBERSORT results and single-cell analysis, which showed that CD53 was closely associated with monocytes, macrophages, neutrophils, and T cells. These findings provide a mechanistic basis for future studies examining whether CD53-positive immune-cell states regulate antigen processing, inflammatory signaling, or antitumor immune responses in LUAD. Regulatory and drug–gene network analyses suggested that the model genes may occupy central molecular positions and provided hypothesis-generating clues for future drug-related experimental studies rather than direct evidence of therapeutic applicability (Truong et al., 2024; Li et al., 2023b).

Our single-cell analysis further refined the interpretation of CD53 from the perspective of tumor heterogeneity. Because bulk transcriptomic data cannot distinguish whether altered CD53 expression is driven by malignant epithelial cells or by changes in immune-cell composition, we incorporated single-cell RNA-seq analysis to determine the cellular distribution of CD53 in the LUAD microenvironment, consistent with recent integrative single-cell and bulk transcriptomic strategies for resolving tumor microenvironmental heterogeneity (Zhang et al., 2024). The results showed that CD53 was predominantly expressed in immune cell populations, particularly T cells, macrophages, and monocytes, whereas its expression was relatively low in epithelial and stromal cell populations. These findings suggest that CD53 is more likely to reflect immune-cell infiltration and immune microenvironmental remodeling than tumor-cell-intrinsic expression alone. This observation is consistent with the known immunoregulatory function of CD53 and supports the immune infiltration results obtained from CIBERSORT.

Importantly, these single-cell findings help explain the apparent complexity of CD53 expression in LUAD. Although CD53 was downregulated in bulk LUAD tissues and clinical specimens, its single-cell distribution suggests that this reduction may be related to altered abundance, localization, or activation states of CD53-positive immune cells within the tumor microenvironment. Therefore, CD53 should be interpreted as an immune-microenvironment-associated biomarker rather than a purely tumor epithelial marker. Recent studies have shown that integrating single-cell and bulk transcriptomic analyses can reveal tumor microenvironmental heterogeneity, identify immune cell states linked to therapeutic stratification, and improve the prediction of immune checkpoint inhibitor response (Lai et al., 2025). Future studies integrating larger LUAD single-cell cohorts, spatial transcriptomics, multiplex immunofluorescence, and treatment-response datasets will be required to determine whether CD53-positive immune-cell states can predict immunotherapy efficacy or other therapeutic outcomes.

Genetic analyses revealed limited colocalization between eQTLs and LUAD GWAS hits, while SMR identified CD53 and HCLS1 as significant downregulated candidates. Therefore, these genetic findings should be interpreted as hypothesis-generating evidence rather than definitive proof of causality. These findings were corroborated by IHC and PCR validation in 30 paired LUAD and adjacent normal tissue samples and by survival analyses, linking low CD53 expression with poor prognosis. The public TCGA-LUAD clinicopathological analysis further strengthened the clinical interpretation of CD53. In addition to the expression validation in clinical specimens, the TCGA-LUAD analysis showed that low CD53 expression was associated with a higher proportion of deceased patients, and continuous CD53 expression was significantly lower in deceased patients than in alive patients. These findings are consistent with the Kaplan–Meier survival analysis and support the potential prognostic relevance of CD53 in LUAD. However, differentiation degree and treatment-response information were not uniformly available in the TCGA-LUAD clinical annotation or in our in-house cohort; therefore, these parameters could not be reliably analyzed. Larger multicenter cohorts with complete pathological, follow-up, and treatment-response information are still required to further validate the clinicopathological and therapeutic relevance of CD53.

The drug–gene network analysis should be interpreted cautiously. Although Medroxyprogesterone acetate and several other compounds were predicted to be associated with the model genes, these findings were derived entirely from computational drug–gene interaction analysis. Therefore, they should be regarded as hypothesis-generating candidates rather than evidence of therapeutic efficacy in LUAD. At present, we have not performed *in vitro* or *in vivo* experiments to validate whether these compounds can regulate CD53-related pathways or inhibit LUAD progression. Future studies should evaluate the predicted compounds using LUAD cell models, immune-cell co-culture systems, organoids, and animal models before any translational or clinical significance can be inferred.

Although CD53 was consistently downregulated in LUAD tissues and was associated with poorer prognosis, its direct biological function in LUAD remains to be experimentally validated. Therefore, CD53 should be interpreted primarily as a candidate immune-microenvironment-associated biomarker, while its potential tumor-suppressive function remains to be verified through future functional studies.

Several limitations should be acknowledged. First, although CD53 showed prognostic relevance in survival analysis, our prognostic assessment was based on a single gene. Single-gene prognostic markers may be less robust and less generalizable than multi-gene or gene-pair-based signatures, particularly across different platforms, cohorts, and clinical settings. Therefore, CD53 should be interpreted as a candidate immune-associated prognostic biomarker rather than a standalone clinical prognostic model. Future studies should integrate CD53 with other immune-related genes, clinicopathological variables, and treatment-response information to construct and externally validate more robust prognostic models. In particular, gene-pair-based approaches may reduce platform-dependent normalization bias by comparing the relative expression ranking between two genes within the same sample. A previous study developed and validated an immune-related gene-pair signature for three urologic cancers and demonstrated its potential for recurrence prediction across multiple cohorts, providing a useful methodological framework for future LUAD prognostic modeling (Xie et al., 2022). Second, the KM survival analysis used expression-based grouping to compare high- and low-CD53 expression groups. Although this approach provides an intuitive visualization of prognostic differences, the use of different cutoffs across cohorts may compromise standardization and limit generalizability. The selection of cut-points for continuous biomarkers can substantially influence survival analysis results, and dichotomizing a continuous variable may also lead to information loss or cutoff-dependent conclusions (Tustumi, 2022). Therefore, future studies should derive a prespecified fixed cutoff from a large, independent training cohort and then apply the same cutoff to external validation cohorts. Alternatively, CD53 expression should be modeled as a continuous variable in Cox regression or flexible survival models, with sensitivity analyses performed to evaluate the robustness of its prognostic association. Third, the present study did not include cell functional experiments or animal model validation to directly determine the biological function and regulatory mechanism of CD53 in LUAD. Although CD53 was consistently downregulated in LUAD and was associated with immune infiltration and prognosis, these findings are mainly based on transcriptomic integration, immune infiltration estimation, genetic association analysis, public database validation, and clinical expression verification. Therefore, the potential tumor-suppressive or immune-regulatory role of CD53 should be interpreted cautiously. Future studies should perform gain- and loss-of-function experiments in LUAD cell lines to assess the effects of CD53 on proliferation, apoptosis, migration, invasion, epithelial–mesenchymal transition, and immune-related signaling. In addition, *in vivo* tumor formation and metastasis models will be required to clarify whether and how CD53 regulates LUAD progression.

Additional limitations include the single-center nature and incomplete clinicopathological annotation of the in-house validation cohort, ancestry and tissue specificity constraints in genetic datasets, and the computational nature of network and drug predictions (Brattoli et al., 2024; Chiu et al., 2019). In particular, the drug–gene network analysis was based on public database-derived associations and did not include experimental validation. Therefore, the predicted compounds should not be interpreted as clinically applicable therapeutic agents, and their efficacy and mechanisms require further validation in cellular, animal, and eventually clinical studies. Mechanistic studies are needed to define how CD53 modulates immune infiltration and tumor progression (Dunlock et al., 2022). Future work should focus on functional characterization of CD53, expanded clinical validation, single-cell and eQTL analyses, and experimental evaluation of candidate therapeutics (Sim et al., 2025).

In conclusion, CD53 emerges as a promising immune-associated biomarker in LUAD, supported by multi-omic and clinical evidence. Our findings provide a foundation for further mechanistic studies and for the experimental validation of CD53-related diagnostic, prognostic, and therapeutic hypotheses (Xia et al., 2025).

## 5 Conclusion

This study identifies CD53 as a core immune-related gene consistently downregulated in bulk LUAD transcriptomic datasets through multi-cohort integration and machine learning. CD53 expression correlates with key immune cell populations, suggesting its involvement in LUAD immune microenvironment remodeling. Single-cell analysis further demonstrated that CD53 is predominantly localized to immune cell populations, especially T cells, macrophages, and monocytes, rather than epithelial cells. Immunohistochemistry and PCR validation in LUAD patient samples confirmed reduced CD53 expression in tumor tissues, and public TCGA-LUAD analysis further supported its clinicopathological and prognostic relevance. These findings highlight CD53 as a candidate immune-microenvironment-associated diagnostic and prognostic biomarker in LUAD, while its potential tumor-suppressive function requires further validation through cell functional assays and animal experiments.

## Data Availability

The original contributions presented in the study are included in the article/Supplementary Material, further inquiries can be directed to the corresponding authors.
