# Supplementary figures and images for: Systems-level analyses and clinical validation highlight CD53 as a diagnostic and prognostic marker in lung adenocarcinoma
Source: Front Cell Dev Biol. 2026 Jun 11;14:1806566. doi: 10.3389/fcell.2026.1806566 (PMC13293822; doi:10.3389/fcell.2026.1806566)

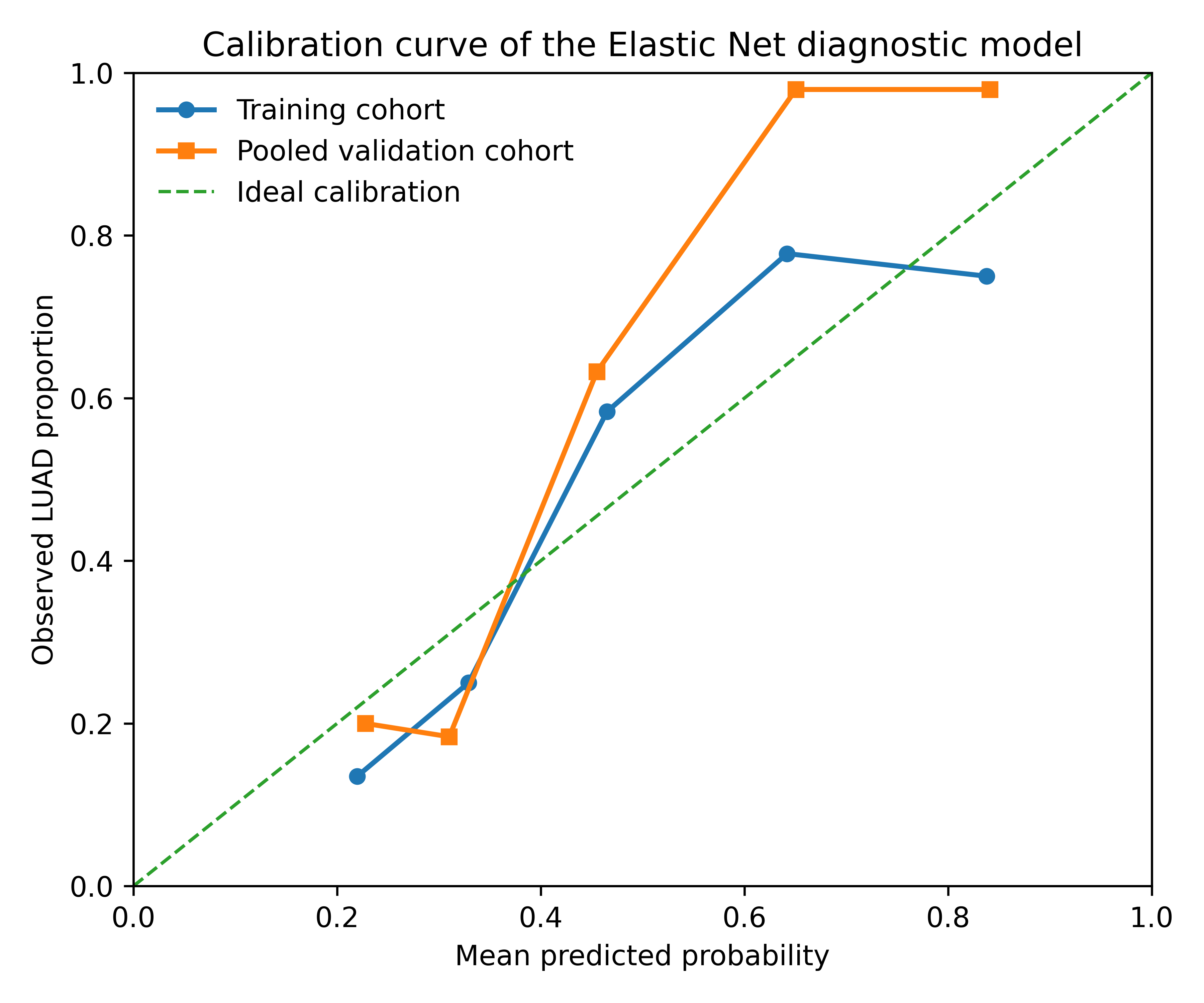

Supplement: Supplementary file 1 [file Image1.png]
